# Intraoperative Image Guidance in Neurosurgery: Development, Current Indications, and Future Trends

**DOI:** 10.1155/2012/197364

**Published:** 2012-05-08

**Authors:** Chris Schulz, Stephan Waldeck, Uwe Max Mauer

**Affiliations:** ^1^Department of Neurosurgery, German Federal Armed Forces Hospital, 89081 Ulm, Germany; ^2^Department of Radiology, German Federal Armed Forces Central Hospital, 56072 Koblenz, Germany

## Abstract

*Introduction*. As minimally invasive surgery becomes the standard of care in neurosurgery, it is imperative that surgeons become skilled in the use of image-guided techniques. The development of image-guided neurosurgery represents a substantial improvement in the microsurgical treatment of tumors, vascular malformations, and other intracranial lesions. *Objective*. There have been numerous advances in neurosurgery which have aided the neurosurgeon to achieve accurate removal of pathological tissue with minimal disruption of surrounding healthy neuronal matter including the development of microsurgical, endoscopic, and endovascular techniques. Neuronavigation systems and intraoperative imaging should improve success in cranial neurosurgery. Additional functional imaging modalities such as PET, SPECT, DTI (for fiber tracking), and fMRI can now be used in order to reduce neurological deficits resulting from surgery; however the positive long-term effect remains questionable for many indications. *Method*. PubMed database search using the search term “image guided neurosurgery.” More than 1400 articles were published during the last 25 years. The abstracts were scanned for prospective comparative trials. *Results and Conclusion*. 14 comparative trials are published. To date significant data amount show advantages in intraoperative accuracy influencing the perioperative morbidity and long-term outcome only for cerebral glioma surgery.

## 1. Background

**Figure 1:**
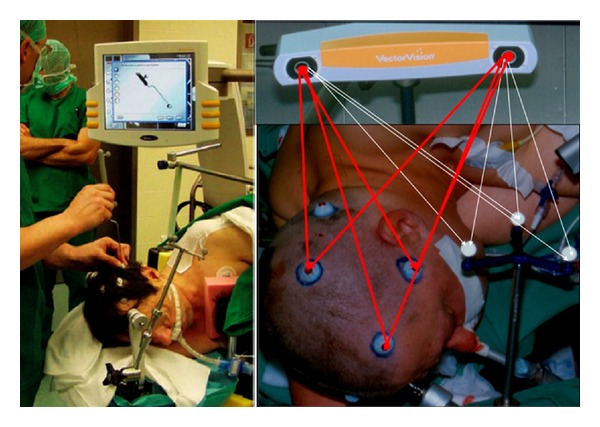
Typical preoperative registration using skin markers (so-called fiducials) and an optical camera system (BrainLab Vector Vision 2) correlating the presurgical image data sets to three-armed-star allowing intraoperative orientation of tracking devices in the surgical site.

**Figure 2:**
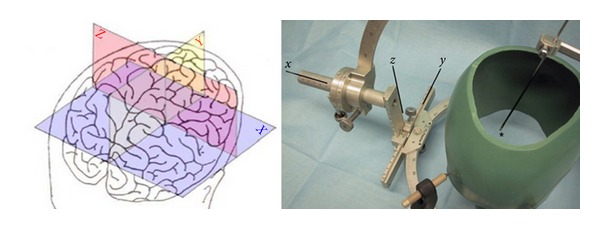
Sketch of a cartesian coordinate system overlaid to a surface sketch of the human brain. Every point within this 3D volume data set is clearly definable by a combination of three coordinates as shown by the skull model fixed to a frame-based sterotaxy system (Zamorano-Dujovny-Apparatus).

**Figure 3:**
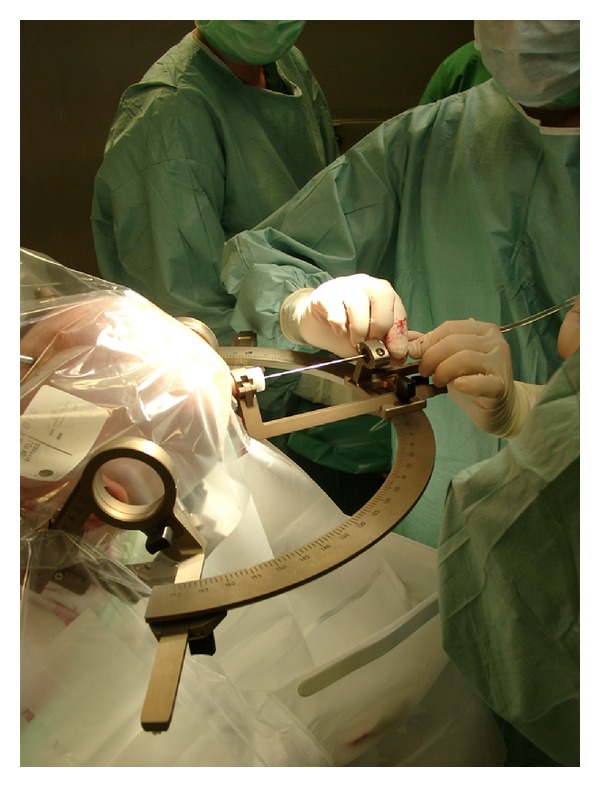
Intraoperative circumstances during intracranial stereotaxy for taking a brain tumor biopsy (using a Leksell Stereotactic System).

The key step for the use of neuronavigational systems is the generation of 3D preoperative image data merged with the patient's anatomy by registration (Figure 1). Once registration is accurate, the surgeon can work in the mathematical space (cartesian coordinate system) of the brain image that is the same as physical space under optimum conditions (Figure 2). The need for image guidance during neurosurgical operations has always been a concern for neurosurgeons and has evolved through several steps over the last 60 years. *Frame-based* navigational systems are the most traditional guidance option (also known as stereotaxy) and have the advantage of being extremely accurate because a rigid head frame is fixed to the skull. Disadvantages include patient discomfort during frame placement, time taken to calculate the trajectory, and inability to project or image where the biopsy probe is. In fact it is a“blind” procedure via a small burr hole without having control over the stereotactic pathway and any complications (bleeding or missing the preplanned aim) during the surgery (Figure 3).

After the era of X-ray view boxes, technological evolution permitted the use of real-time imaging such as fluoroscopy and intraoperative ultrasound. The introduction of computed tomography (CT) in the 1970s and magnetic resonance imaging (MRI) in the 1990s led to neuronavigation systems for surgical planning and working. *Frameless neuronavigational* systems in contrast to a frame-based devices track the movement of surgical instruments in space by optical, ultrasonic, or electromagnetic sensors. Their relative position to the lesion can be projected from preoperative imaging but not in an intraoperative real-time modus. The rapid development of navigational devices has provided the neurosurgeon with an unprecedented degree of surgical accuracy and precision for the planning as well as performance of a large variety of neurosurgical procedures. In this context, the development of image-guided neurosurgery represents a substantial improvement in the microsurgical treatment of tumors, vascular malformations, and other intracranial lesions. Despite the wide applicability and many fascinating aspects of image-guided navigation systems, a major drawback of this technology became apparent right from the beginning of its implementation in neurosurgical operations. All these neuronavigational systems use images, mainly CT or MRI pictures, acquired *pre*operatively, on which the planning of the operative procedure as well as its *intra*operative performance is based. They, therefore, have several potential sources of error. Registration (using skin markers or anatomical landmarks) may be inaccurate mostly because of scalp movement, geometrical distortion in the images, and movement of the patient with respect to the system during surgery. Another relevant source of inaccuracy is the so-called brain shift (movement of the brain relative to the cranium between the time of scanning and the time of surgery). Due to these sources of error, the usefulness of surgical navigation may diminish during the surgical procedure. As dynamic changes of the surgical field regularly occur during the surgical procedure, the surgeon is faced with a continuously changing intraoperative field for which the preoperative data does not provide any information. It is clear that only *intra*operatively acquired images will provide the neurosurgeon with the online information he needs to perform real intraoperative image-guided surgery. A number of high-tech tools for use during neurosurgical procedures have been developed in recent years, like intraoperative navigated ultrasound and dedicated moveable intraoperative CT units. However, MRI currently is and definitely will be in the future the superior imaging method for intraoperative image guidance.

Whether image guidance techniques are advantageous remains questionable for the last decades. Valuable information from numerous large prospective randomized trials are not available to date and a definite effect on long-term outcome needs yet to be proven. Therefore, the surgeon is left to decide on a case-by-case basis whether to perform surgery with or without neuronavigation. Beside this there will be an increasing pressure on neurosurgeons to justify the costs involved by showing that patients will actually benefit from complex image-guided treatments.

## 2. Data Source/Study Selection

A PubMed literature search using the terms“image guided neurosurgery” was performed. More than 1400 articles were published during the last 25 years. All the abstracts were scanned for interesting historical and technical notes but especially for comparative clinical trials (either retro- or prospective), which is the focus of this overview.

## 3. Results

Image guidance in neurosurgery is today a frequently used tool for operations of many brain lesions, mostly for small and deeply subcortical located brain tumors or cavernomas [1]. The increased efficacy and safety was quickly recognized for vascular malformations, for complex skull base surgery and, for endoscopic operations and so-called single burr-hole-maneuvers like biopsy, catheter placement, cyst aspiration, and foreign body removal [2–4]. Some of the assumed advantages of intraoperative imaging to intracranial surgery are as follows:

- detailed preoperative planning (positioning, approach, and trajectory);
- integration/fusion of MRI/CT images and functional data (angiography, PET, SPECT, DTI, fMRI, and electrophysiology);
- limited surgical exposure;
- more precision in approach;
- more precision in biopsy;
- more precision in catheter/electrode placement;
- guidance and control of tumor resection/foreign body removal;
- optimizing the size of intraoperatively buildt cranial reconstruction devices;
- monitoring complications (using intraoperative MRI or ultrasound).

The most important step in the development of cranial neuronavigation was the availability of intraoperative MRI (ioMRI) which has led to a variety of differently designed systems and concepts [5]. Nowadays ioMRI allows neurosurgeons not only to increase the extent of tumor resection and to preserve eloquent areas or white matter tracts but it also provides physiological and biological data of the brain and tumor tissue as well as the intraoperative detection of complications [6–8]. The most relevant advantage is the possibility to have the registration directly in the OR (with reduced registration error) and to perform a repeated registration following dynamic changes of the intraoperative field (viz., brain shift). Despite its increasing usefulness, there are some disadvantages to ioMR (very expansive, time consuming, only nonferromagnetic instruments are possible in the magnetic field, not possible for patients with ferromagnetic implants).

Though neuronavigation has become a routinely used addition to the neurosurgical armamentarium, its impact on surgical results has not yet been examined sufficiently for all indications. The most important and best examined use of neuronavigation and ioMRI is for cerebral glioma surgery. To achieve total tumor resection is the primary goal of these operations, but its positive effect on survival of the patients was controversially discussed for several decades [9, 10]. However, more and more data now show the positive benefit from surgery on low- and high-grade glioma as radical as possible, even in eloquent brain areas [11–18]. Taken together there are 14 comparative studies concerning the effect of using neuronavigation and ioMRI in glioma surgery. In a randomized controlled study the mean amount of residual tumor tissue was 28.9% for standard surgery and 13.8% for surgery involving neuronavigation (without ioMRI). The corresponding mean amounts of residual contrast-enhancing tumor tissue were 29.2 and 24.4%, respectively, and these differences were not significant [19]. In contrast Wirtz et al. found that the operating times were identical in two comparative groups (with versus without neuronavigation), while preparation times were 30.4 min longer with navigation. Radiological radicality was achieved in 31% of navigation cases versus 19% in conventional operations. The absolute and relative residual tumor volumes were significantly lower with neuronavigation and radical tumor resection was associated with a highly significant prolongation in survival (median 18.3 versus 10.3 months). Survival was longer in patients operated on using neuronavigation (median 13.4 versus 11.1 months) [20]. The use of neuronavigation combined with ioMRI is relevantly influencing the operative strategy in 30% to 50% of glioma surgeries with the result of a more radical resection and improved neurological function compared to traditional or standard navigated operative manner [21]. Intraoperative resection control using ioMRI led to further tumor resection in 28.6% of patients with contrast-enhancing tumors and in 47.6% of patients with noncontrast-enhancing tumors. In contrast-enhancing tumors, further resection led to an increased rate of complete tumor resection (71.2 versus 52.4%) [22]. Muragaki et al. showed that compared to a control group (operated on without ioMRI), the resection rate in the study group (operated on using ioMRI) was significantly higher (91%, versus 95%), whereas residual tumour volume was significantly smaller (1.7 mL versus 0.025 mL) [23]. In another study ioMRI showed primary complete resection in 27% of all glioma patients. In 41% of all patients the resection was extended owing to intraoperative MRI increasing the percentage of complete resections to 40% [24–26]. These positive radiological outcome data are now supported by a prospective randomized controlled study [27, 28]. But whether these improvements really affect the long-term outcome of patients suffering for cerebral glioma was still a matter of debate [29]. Today several current data show that overall survival is significantly prolonged, progression-free survival is increased and neurological function is more often preserved [30].

In a retrospective single center analysis the median survival periods of patients receiving gross total resection for malignant astrocytoma (versus partial resection) and neuronavigation (versus no neuronavigation) were 16 (versus 9) months and 16 (versus 10) months, respectively. The percentage of a gross total resection was significantly higher in the neuronavigation group compared to that in the no-navigation group (64.3% versus 38.2%). Neurological deterioration occurred in 4 of 42 (9.5%) and in 6 of 34 (17.6%) patients after surgery with neuronavigation and surgery without neuronavigation [31]. In another study the average operating time using ioMRI was 5.1 hours and was significantly longer than in the conventionally treated patients (3.4 hours). The mean overall survival time for the 32 glioma patients in the study group was 14.5 months compared to 12.1 months for the retrospective matched control group [32]. Patients after subtotal resection of a low-grade glioma were at 1.4 times the risk of recurrence and at 4.9 times the risk of death relative to patients who underwent gross total resection. The 1-year, 2-year, and 5-year age-adjusted death rates for patients who underwent surgical resection using ioMRI guidance were 1.9%, 3.6%, and 17.6%, respectively, which is significantly lower than the rates reported in former publications without the use of ioMRI [33, 34]. When analyzing survival of patients with glioblastoma, patients undergoing complete tumor resection using ioMRI did significantly better than patients with residual tumor (50% survival rate at 57.8 weeks versus 33.8 weeks) [35]. Another prospective comparative analysis showed a median survival time for patients in whom ioMRI had been used of 20.37 months compared to 10.3 months in the cohort who had undergone conventional microsurgical removal [36]. Wu et al. showed in prospective controlled study on 238 glioma patients with involvement of the pyramidal tract a significant improved rate of gross total resection using ioMRI combined with DTI fiber tracking (74.4 versus 33.3%) in high-grade glioma. However, there was no significant difference of low-grade glioma resection between the two groups. Postoperative motor deterioration occurred in 32.8% of control cases, whereas it occurred in only 15.3% of the study cases. The 6-month Karnofsky Performance Scale score of study cases was significantly higher than that of control cases. For 81 malignant glioma, the median survival of study cases was 21.2 months compared with 14.0 months of control cases. The estimated hazard ratio for the effect of DTI-based functional neuronavigation was 0.570, representing a 43.0% reduction in the risk of death [37]. These data support the findings from Reithmeier et al., who examined 42 patients [38]. Senft et al. found in their study More patients in the intraoperative MRI group to have complete tumour resection (23 [96%] of 24 patients) than did in the control group (17 [68%] of 25, *P* = 0.023). Postoperative rates of new neurological deficits did not differ between patients in the intraoperative MRI group (three [13%] of 24) and controls (two [8%] of 25, *P* = 1.0). No patient for whom use of intraoperative MRI led to continued resection of residual tumour had neurological deterioration [27, 28]. In 25.9% of all cases examined by Kuhnt et al., additional tumor mass was removed as a result of iMRI. This led to complete tumor resection in 20 cases, increasing the rate of gross-total removal from 31.7% to 38.6%. In 56 patients, additional but incomplete resection was performed because of the close location to eloquent brain areas. Volumetric analysis showed a significantly (*P* < 0.01) reduced mean percentage of tumor volume following additional further resection after iMRI and they concluded that MRI in conjunction with multimodal navigation and an intraoperative updating procedure enlarges tumor-volume reduction in glioma surgery significantly without higher postoperative morbidity [16–18].

Although the number of prospective randomized studies is low at the moment, the controversies to use image-guided neurosurgery for the resection of cerebral glioma (either low or high graded) increasingly are unjustified. That is what Kubben et al. concluded in their review, too [39]. The sufficiency we have now for glioma surgery is not so high for other tumor entities (for instance meningioma or hypophyseal adenoma) as well as for vascular malformations [40, 41]. Beside this, no prospective randomized data are available concerning the cost effectiveness of neuronavigation.

## 4. Future Directions and Conclusions

### 4.1. Future Trends in Clinical Applications and Imaging Techniques

Frameless stereotactic neurosurgery is increasingly being used for the biopsy of intracranial tumors and the resection of deep-seated lesions where reliance on surface anatomic landmarks can be misleading, as well as in movement disorders, psychiatric disorders, seizure disorders, and chronic refractory pain [42–45]. Nascent biological approaches, including gene therapy and stem-cell and tissue transplants for movement disorders, also utilize neuronavigational techniques. These procedures are complex and involve understanding of the basic principles and factors affecting neuronavigation.

**Figure 4:**
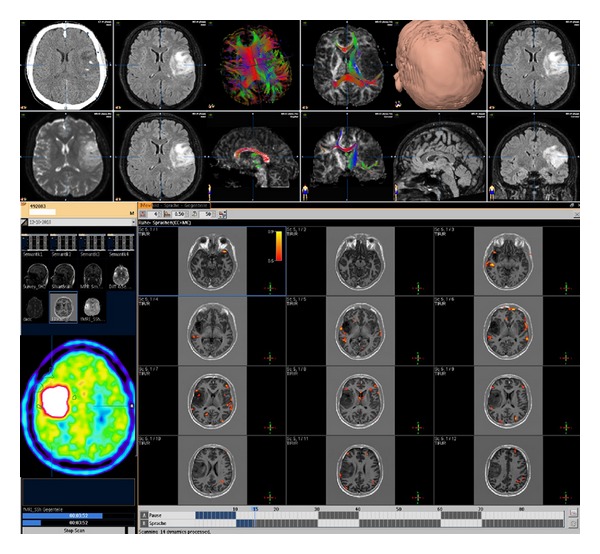
3D volume data set combining CT images overlayed with FLAIR weigthed MRI, DTI tractography, and fMRI to define speech areas in a patient with a left parietal low grade astrocytoma.

**Figure 5:**
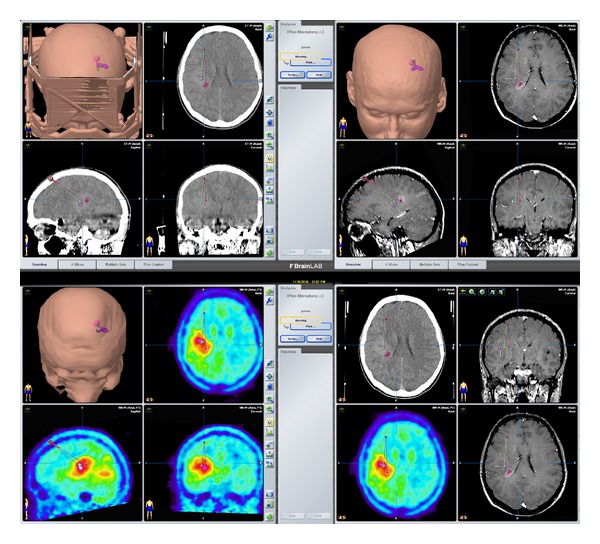
Presurgical navigational overlay of CT, MRI, and FDG-PET in a patient prepared for a stereotactic biopsy. The CT images showed only minor changes, the T1 weighted contrast-enhanced MRI showed a very small contrast affine region but a large hypermetabolic lesion in the left parietal brain was revealed by PET, helping to define the best biopsy aim and trajectory.

One of the goals of brain surgery is to avoid damage to eloquent cortex and subcortical white matter. Diffusion tractography remains the only noninvasive method capable of segmenting the subcortical course of a white matter tract and has rapidly become an important clinical tool that can delineate functionally important white matter tracts for surgical planning [16–18, 46], (Figure 4). Since the advent of neuronavigation devices, these systems have been used mainly to acquire information concerning intraoperative anatomy [24–26, 47]. Functional neuronavigation (the combination of image-guided neurosurgery, functional MR imaging, nuclear medicine imaging, and physiological examinations) is a new method that allows fast orientation of the relation of the lesion to functional anatomy by incorporation of localization data of the sensorimotor cortex as well as language, and memory areas into neuronavigation systems, allowing the identification of new anatomical targets and clinical indications. In the future, coregistration of high resolution anatomic and neurophysiological data from multiple complementary sources will be used to plan more neurosurgical procedures, including minimally invasive procedures (Figure 5). Along the way, new insights on fundamental processes such as the biology of tumors and brain plasticity are likely to be revealed. This is a next step in an evolving process of integrating data other than anatomical information into the surgical site [48–51].

### 4.2. Future Trends in Intraoperative Imaging and Remote Operating Systems

The intraoperative use of MR imaging in neurosurgery has just started and future developments in this technology will surely add to the rapidly evolving field of MRI-guided neurosurgery [52]. Preoperative 3D-imaging data in addition to overlayed intraoperative conditions do allow new applications in planning, simulating, and working strategies, like in a virtual or an augmented reality surgery [53, 54]. Requirements of virtual reality for surgery include registration of patient data with atlases and the ability to coregister multimodal patient data. For use over extended periods, which is often needed in surgery, the style of user interaction should be natural, comfortable, and easy to use. One promising area where VR could make a contribution is in remote diagnostics, where two surgeons can confer on a particular case, each experiencing the same 3D visualisation, although located in different places. The other, often discussed, main applications are for remote operations, either through robotic surgery, or through assistance to another remote surgeon. These possibilities resulting in telemedicine applications like teleconsultation or teleassisting are of interest especially for inexperienced surgeons in the military setting or in developmental regions [55]. The main problem here is the network delay, since almost immediate interactivity is required. Even the small delay introduced by the use of satellite communication is unacceptable in remote neurosurgery.

Robots are used more routinely nonremotely, for precision in carrying out certain procedures, such as skull base approaching craniotomies [56]. The types of operation to which robots are applied in this way are usually high volume, repeated procedures. In addition to improved accuracy, major cost savings can be produced. A relatively new development is to use surgeon-controlled robots to carry out, by key-hole methods, operations which previously required open surgery. VR becomes important here in providing a detailed 3D view to guide the surgeon in carrying out the operation via extremely small robotic instruments. Operations, such as brain biopsy, cyst aspiration, catheter placement, and electrode implantation in functional regions could be carried out in this way with reduced trauma and recovery time for the patient. The technical possibility already exists for unsupervised robots to carry out surgery, but much ethical and legal debate and legislation will be needed before this could be put into practice [53, 57, 58].

Image-guided surgery is technically demanding and a learning curve has to be completed. Minor inaccuracies in the handling of the technical equipment might translate into major surgical errors. These errors, once implemented are systemic errors that propagate through the whole procedure. Navigation systems might become an important cornerstone in neurosurgery education. While today neurosurgery education takes place in the OR and to a lesser degree in cadaver and hands-on workshops the systematic development of education and training modules using navigation technology might offer a new way to develop and improve the perceptive and locomotive capabilities necessary to perform surgery on an organ that has a complex three-dimensional anatomy which is to a large extent hidden from direct visual perception [59]. However, while the clinical applications of image-guidance systems have reached a high standard, the opportunities image-guidance systems offer as educational tools have not been investigated systematically to date.

### 4.3. Conclusions

Since the integration of 3D image processing and real-time tracking of smart tools, the feasibility of image-guided approaches of many application in cranial surgery has been proven. Currently, the question whether the implementation of an extremely costly high-tech tool like the MRI in the neurosurgical operating room represents a technical overkill restricted to only a very small number of high-class neurosurgical centers, or whether it is and will be a major breakthrough in modern neurosurgery cannot be clearly answered, possibly except for the surgery on cerebral glioma. As an increasing number of ioMRI units will be installed in neurosurgical operating theatres worldwide, one have to await the increasing scientific evaluation of this technology, which will help to define the future role of neuronavigation and its integrated functional imaging and physiological data.

## Abbreviations

MRI: Magnetic resonance tomography
fMRI: Functional magnetic resonance tomography
ioMRI: Intraoperative magnetic resonance tomography
CT: Computed tomography
PET: Positron emission tomography
DTI: Diffusion tensor imaging
VR: Virtual reality.
